# Toripalimab combined with lenvatinib and GEMOX is a promising regimen as first-line treatment for advanced intrahepatic cholangiocarcinoma: a single-center, single-arm, phase 2 study

**DOI:** 10.1038/s41392-023-01317-7

**Published:** 2023-03-17

**Authors:** Guo-Ming Shi, Xiao-Yong Huang, Dong Wu, Hui-Chuan Sun, Fei Liang, Yuan Ji, Yi Chen, Guo-Huan Yang, Jia-Cheng Lu, Xian-Long Meng, Xin-Ying Wang, Lei Sun, Ning-Ling Ge, Xiao-Wu Huang, Shuang-Jian Qiu, Xin-Rong Yang, Qiang Gao, Yi-Feng He, Yang Xu, Jian Sun, Zheng-Gang Ren, Jia Fan, Jian Zhou

**Affiliations:** 1 Department of Liver Surgery and Transplantation, Liver Cancer Institute, Zhongshan Hospital, Fudan University, Shanghai, China; 2 Department of Radiology, Zhongshan Hospital, Fudan University, Shanghai, China; 3 Department of Statistics, Zhongshan Hospital, Fudan University, Shanghai, China; 4 Department of Pathology, Zhongshan Hospital, Fudan University, Shanghai, China; 5 Department of Hepatic Oncology, Liver Cancer Institute, Zhongshan Hospital, Fudan University, Shanghai, China; 6 Tianjin Medical Laboratory, BGI-Tianjin, BGI Shenzhen, Tianjin, China

**Keywords:** Drug development, Gastrointestinal cancer

## Abstract

Advanced intrahepatic cholangiocarcinoma (ICC) has a dismal prognosis. Here, we report the efficacy and safety of combining toripalimab, lenvatinib, and gemcitabine plus oxaliplatin (GEMOX) as first-line therapy for advanced ICC. Thirty patients with pathologically confirmed advanced ICC received intravenous gemcitabine (1 g/m^2^) on Days 1 and 8 and oxaliplatin (85 mg/m^2^) Q3W for six cycles along with intravenous toripalimab (240 mg) Q3W and oral lenvatinib (8 mg) once daily for one year. The expression of programmed death-ligand 1 (PD-L1) and genetic status was investigated in paraffin-embedded tissues using immunohistochemistry and whole-exome sequencing (WES) analysis. The primary endpoint was the objective response rate (ORR). Secondary outcomes included safety, overall survival (OS), progression-free survival (PFS), disease control rate (DCR) and duration of response (DoR). As of July 1, 2022, the median follow-up time was 23.5 months, and the ORR was 80%. Twenty-three patients achieved partial response, and one achieved complete response. Patients (21/30) with DNA damage response (DDR)-related gene mutations showed a higher ORR, while patients (14/30) with tumor area positivity ≥1 (PD-L1 staining) showed a trend of high ORR, but without significant difference. The median OS, PFS, and DoR were 22.5, 10.2, and 11.0 months, respectively. The DCR was 93.3%. Further, 56.7% of patients experienced manageable grade ≥3 adverse events (AEs), commonly neutropenia (40.0%) and leukocytopenia (23.3%). In conclusion, toripalimab plus lenvatinib and GEMOX are promising first-line regimens for the treatment of advanced ICC. A phase-III, multicenter, double-blinded, randomized study to validate our findings was approved by the National Medical Products Administration (NMPA, No. 2021LP01825).

**Trial registration Clinical trials**: NCT03951597.

## Introduction

Biliary tract cancer (BTC) is a heterogeneous spectrum of high aggressive adenocarcinomas including gallbladder cancer, intrahepatic cholangiocarcinoma (ICC), and extrahepatic cholangiocarcinoma (ECC).^1^ The incidence of cholangiocarcinoma is low in North America and western Europe (0.35–2 cases per 100,000, annually) but up to 40 times higher in Thailand and China.^2^ ICC ranks as the second most common primary liver neoplasm with rising incidence globally.^1^

Since ICC is quite different from other BTC in molecular phenotype, immune microenvironment and prognosis, it is of great significance to explore new treatments of pure ICC.^1^ Currently, surgery is the first option for early stage ICC. Unfortunately, 60–88% of ICC cases are diagnosed at a late stage,^1^ and unresectable ICC remains dismal survival (median <5 months without treatment^3^ or approximately 1 year with gemcitabine-based chemotherapy).^4^ The current preferred first-line chemotherapy for locally advanced or metastatic BTC, including ICC, is gemcitabine plus cisplatin (GEMCIS), which yields a median overall survival (OS) of 11.7 months.^4^ Its objective response rate (ORR) in bile duct and ampullary tumors is 19.0%, while its ORR is 37.7% in gallbladder tumors.^4^ For patients in Asia, oxaliplatin plus gemcitabine (GEMOX) is another common treatment regimen. Data indicate that GEMOX (699 patients from 15 trials) caused less grade 3 and 4 adverse events (AEs) including asthenia, diarrhea, liver toxicity, and hematological toxicity than GEMCIS (771 patients from 18 trials) but yielded a similar median OS (9.5 months vs. 9.7 months, respectively) for patients with locally advanced or metastatic BTC.^5^ Further work is necessary to enhance the clinical efficacy of chemotherapy for ICC.^6^

Recent evidence suggests that tumor eradication and improved survival can be promoted by anti-PD-1 antibodies, and blockade of PD-1/PD-L1 pathways reinvigorates antitumor immunity. Monotherapy and combination therapy with anti-PD-(L)1 have achieved an improved tumor response rate and extended survival times for a spectrum of advanced malignancies,^7^ e.g., melanoma, lung cancer, colorectal cancer, head and neck cancer, hepatocellular carcinoma (HCC), and gastric cancer. Lately, TOPAZ-1 trial showed Durvalumab plus GEMCIS provided significant survival benefits for patients with locally advanced or metastatic BTC compared to GEMCIS chemotherapy (median OS: 12.8 months versus 11.5 months).^8^ Thus, this regimen also was recommended as one of preferred first-line therapies for advanced BTC. In cohorts of pure ICC, however, there is no clinical trial reporting improved survival with PD-1 inhibitor monotherapy or combination therapy,^9–11^ but an observational study did find that PD-1/PD-L1 expression profiles could effectively predict ICC clinical prognosis.^12^ Additionally, endogenous antitumor responses, represented as tumor-infiltrating lymphocytes, were observed in all ICC tumors,^13,14^ suggesting that anti-PD-1 antibody therapy may exert clinical benefit for patients with ICC.

Above phenomenon inspired the current study, a single-center, open-label, single-arm, phase 2 investigation to investigate the efficacy and safety of the anti-PD-1 antibody toripalimab in combination with lenvatinib and GEMOX chemotherapy for advanced ICC. Toripalimab is a humanized anti-PD-1 IgG4 monoclonal antibody approved for clinical trials by the US Food and Drug Administration (FDA) and China’s National Medical Products Administration (NMPA).^15,16^ This drug demonstrates promising efficacy and safety profiles for urologic cancer,^17^ melanoma,^17,18^ and gastric cancer.^19^ Lenvatinib is a multikinase inhibitor that targets vascular endothelial growth factor receptor (VEGFR) 1 to 3, fibroblast growth factor receptors (FGFR) 1 to 4, platelet-derived growth factor receptor-a (PDGFRa), RET, and KIT. A high expression level of vascular endothelial growth factor (VEGF) was detected in 53.8% of ICC and was considered to be involved in hematogenic metastasis.^20^ The FGFR signaling pathway is also abnormally activated in ICC and is associated with poor prognosis.^21^ Finally, considering that lenvatinib and chemotherapy regimens can significantly upregulate PD-L1 expression,^22^ using these therapies with anti-PD-1 treatment may significantly enhance their effects. Notably, combined therapy of anti-PD-1 with lenvatinib is reported to be useful for the treatment of several cancer types, and the FDA has approved lenvatinib plus pembrolizumab for treating advanced endometrial cancer and advanced renal cell carcinoma.^23^ Therefore, it is reasonable to combine anti-PD-1 with lenvatinib and GEMOX chemotherapy in ICC. Our findings show a new and promising treatment approach for advanced ICC.

## Results

### Patients

**Fig. 1:**
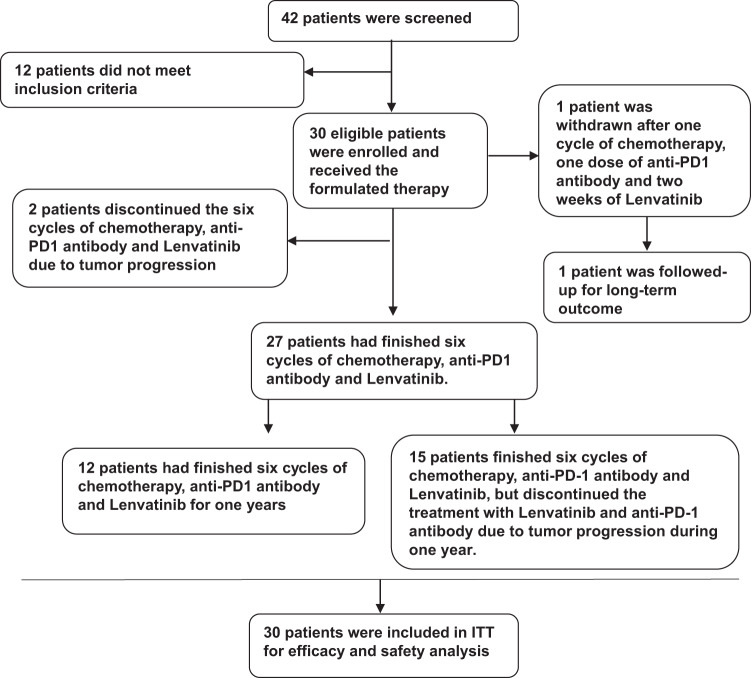
Flowchart for participants’ selection

Forty-two subjects were screened. Twelve patients were excluded from the trial due to refusal of chemotherapy (*n* = 7), pathological inconformity of ICC (*n* = 3), or failure of other inclusion criteria (one patient had jaundice, and one patient was diagnosed with hilar cholangiocarcinoma). Thirty eligible subjects were enrolled and received the formulated therapy (Fig. 1).

Among the 30 patients with advanced ICC, the age ranged from 25 to 73 years old (mean, 56.5 years old), and 63.3% of patients (19/30) were male (Table 1). ECOG evaluation showed that all patients had fully active disease, received no systemic therapy before enrollment, and were at TNM stage IIIA (5/30, 16.7%) or higher (25/30, 83.3%) before treatment. A total of 26.7% of patients showed positive marker of hepatitis B surface antigen (HBsAg) (8/30), 80.0% of patients had hepatitis B core antibody (HBcAb) positive (24/30), and one patient had a history of hepatolithiasis (1/30, 3.3%)—both major risk factors for ICC tumorigenesis. Seventy percent (21/30) of patients had elevated level of carbohydrate antigen 19-9 (CA19-9, ≥37 U/mL).

**Table 1:** Baseline characteristics of 30 patients

Characteristics	All patients (*n* = 30)
*Age, years*	
Mean (range)	56.5 (25–73)
*Sex*	
Male	19 (63.3)
Female	11 (36.7)
*ECOG performance status*	
0	30 (100)
1	0 (0)
*TNM stage at baseline*	
IIIA	5 (16.7)
IIIB	13 (43.3)
IV	12 (40.0)
*History of hepatolithiasis*	
Yes	1 (3.3)
No	29 (96.7)
*HBsAg*	
Positive	8 (26.7)
Negative	22 (73.3)
*HBcAb*	
Positive	24 (80.0)
Negative	6 (20.0)
*Prior systemic therapy*	
Yes	0 (0)
No	30 (100)
*PD-L1 expression (IHC* *≥* *1%*^*a*^*)*	
Positive	14 (46.7)
Negative	16 (53.3)
*CA19-9 (U/mL)*	
≥37	21 (70.0)
<37	9 (30.0)
*DDR-related gene mutation*	
Present	21 (70.0)
Absent	9 (30.0)
*MSI status*	
High	2 (6.7)
Low	3 (10.0)
Stable	25 (83.3)
*BRAF mutation*	
Yes	3 (10.0)
No	27 (90.0)
*FGFR2 fusions/arrangement*	
Yes	1 (3.3)
No	29 (96.7)
*IDH1 mutation*	
Yes	1 (3.3)
No	29 (96.7)
*Tumor mutation burden (Muts/Mb)*	
Median (range)	0.985 (0.08–31.82)

*ECOG* Eastern Cooperative Oncology Group, *HBsAg* hepatitis B surface antigen, *HBcAb* hepatitis B core antibody, *PD-L1* programmed cell death-ligand 1, *CA19-9* carbohydrate antigen 19-9, *DDR* DNA damage response, *MSI* microsatellite instability, *FGFR2* fibroblast growth factor receptor 2, *IDH1* isocitrate dehydrogenase

^a^Tumor area positivity (TAP) ≥ 1% was defined as positive. Proportion of tumor and/or immune cells with PD-L1 staining at any intensity

### Delivery and efficacy of the combination of toripalimab plus lenvatinib and GEMOX

**Fig. 2:**
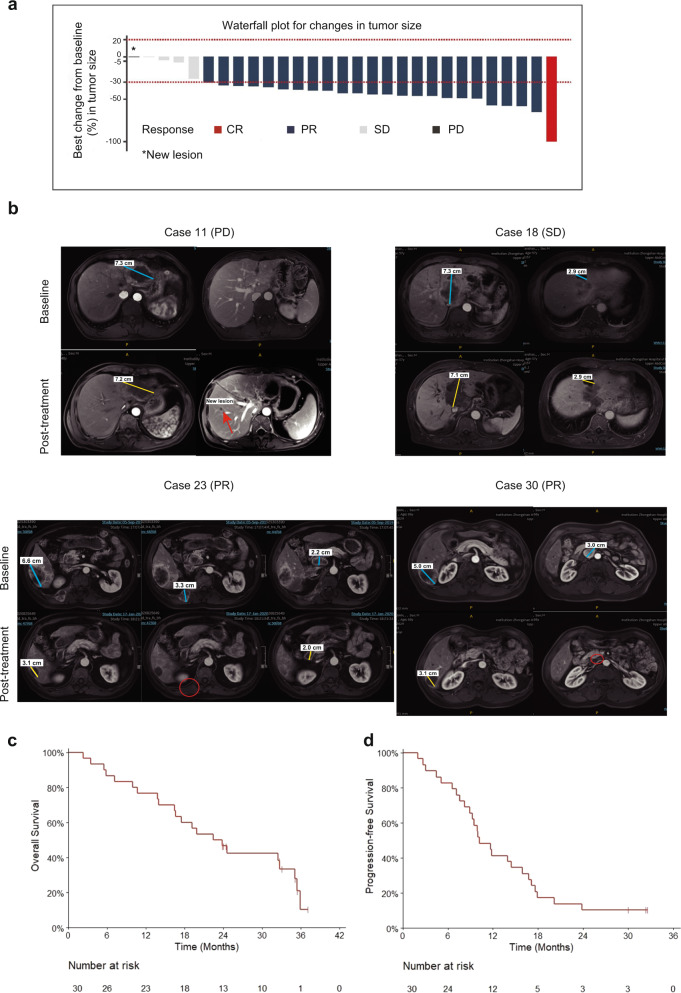
Tumor response to combined therapy of toripalimab combined with lenvatinib and GEMOX. **a** Combined therapy on changes in tumor size for each patient; one patient was not evaluated due to withdrawal of informed consent after one dose of medication. **b** Representative image with different tumor response to the combined therapy (case 11 with PD; case 18 with SD; case 23 with PR, case 30 with PR) (Red arrow in case 11 refers to new lesion after the combined therapy; red circle in case 23 refers to disappear of bone metastasis; and red circle in case 30 refers to disappear of lymph node metastasis.) **c** Kaplan–Meier Survival Curves for estimating the probability of overall survival of the entire cohort; **d** Kaplan–Meier Survival Curves for estimating the probability of progression-free survival of the entire cohort

Thirty patients were included in the intention-to-treat set for analysis of efficacy and safety (Fig. 1). At the data cutoff for the analysis (July 1, 2022), all 30 patients had completed follow-up, and 8 (26.7%) patients survived. Among all patients, the median time of combined treatment of lenvatinib with toripalimab was 10.2 months (range: 1–26.5 months), and 27 patients completed 6 cycles of GEMOX chemotherapy. The dose of GEMOX was modified in 6 patients due to adverse events (AEs), including 2 cases due to leukocytopenia, 4 cases due to thrombocytopenia, sepsis, increased creatinine level or increased total bilirubin. Treatments were delayed in 9 patients due to AEs. Among 9 patients, leukocytopenia was the most common AE and observed in 3 patients. Other AEs, such as thrombocytopenia, fever, sepsis, increased creatinine level increased total bilirubin, and hyperthyroidism, occurred in each case respectively. None discontinued the treatment due to AEs. Notably, investigator review indicated that twenty-three (76.7%) patients achieved partial response (PR) to the combination therapy, four (13.3%) had stable disease (SD), one (3.3%) experienced complete response (CR), one (3.3%) had progressive disease (PD) at 10 weeks after first medication, and one (3.3%) was not evaluated due to withdrawal of informed consent after one dose of medication (Fig. 2a). The best response of each subject is summarized in Supplementary Table 1. In total, 83.3% of patients with TNM stage III (*n* = 18) at baseline achieved PR, and 75% of patients with stage IV (*n* = 12) achieved PR or CR (Table 2 and Supplementary Table 1). The best response rate was 80% (*n* = 24; 95% CI: 61.4–92.3), and the DCR was 93.3% (*n* = 28; 95% CI: 77.9–99.2) (Fig. 2a). Tumor response was assayed using magnetic resonance imaging or computed tomography by the investigator per the Response Evaluation Criteria in Solid Tumors (RECIST) 1.1. Representative cases are shown in Fig. 2b. At the end of follow-up, tumor response was further assessed by two independent radiologists for post hoc analysis. The results showed an ORR of 80% (24/30), similar to that assessed by the investigators. The DCR was 90% (27/30) in the post hoc analysis, and one patient with SD by the investigator’s assessment was defined as PD in the post hoc analysis.

**Table 2:** Summary of treatment efficacy assessed by the investigator

Responses	All patients (*n* = 30)
CR, *n* (%)	1 (3.3)
PR, *n* (%)	23 (76.7)
SD, *n* (%)	4 (13.3)
PD, *n* (%)	1 (3.3)
NE, *n* (%)	1 (3.3)
ORR, *n* (%)	24 (80.0) (95% CI: 61.4–92.3)
DCR, *n* (%)	28 (93.3) (95% CI: 77.9–99.2)
Median DoR	11 months (95% CI: 7.8–15 months)
Median TTR	2.1 months (95% CI: 2.07-2.13months)
Median PFS	10.2 months (95% CI: 9.3–16.8 months)
Median OS	22.5 months (95% CI: 15.6 months–29.3 months)
1-year OS (%)	76.7 (95% CI: 62.9–93.4)
1-year PFS (%)	41.4 (95% CI: 26.8–63.8)
2-year OS (%)	49.8 (95% CI: 34.7–71.4)

*CR* complete response, *PR* partial response, *SD* stable disease, *PD* progression disease, *NE* not evaluable, *ORR* objective response rate, *DCR* disease control rate, *DoR* duration of response, *TTR* time to response, *PFS* progression-free survival, *OS* overall survival, *CI* confidence interval

After a median of 23.5 months (range: 2.4–37.1 months) of follow-up at the data cutoff, the median OS was 22.5 months (95% CI: 15.6–29.3 months). 1- and 2-year survival rates were 76.7% (95% CI: 62.9–93.4) and 49.8% (95% CI: 34.7–71.4), respectively (Table 2 and Fig. 2c). The median PFS was 10.2 months (95% CI, 9.3–16.8 months), and the 1-year PFS rate was 41.4% (95% CI: 26.8–63.8; Table 2 and Fig. 2d). The median time to response (TTR) was 2.1 months (95% CI: 2.07–2.13 months) (the TTR of each patient is listed in Supplementary Table 1). The median DoR was 11.0 months (95% CI, 7.8–15.0 months) (the DoR of each subject is listed in Supplementary Table 1). Radical resection was done in three patients with locally advanced ICC after downgrading, all three of whom survived. Moreover, two patients survived without recurrence.

### Adverse events (AEs)

All AEs are reported in Table 3. The 10 most common (≥50%) AEs were increased AST or ALT levels, thrombocytopenia, anemia, neutropenia, leukocytopenia, abnormal electrocardiogram (ECG), vomiting, nausea, and fatigue. No grade 5 AEs were observed in this study, while 100% (30/30), 56.7% (17/30), and 10% (3/30) of patients experienced grades 2, 3, and 4 AEs, respectively, and these AEs were suspected to be treatment-related. In present study, the most common grade 3 or higher treatment-emergent adverse events (TEAEs) included neutropenia (*n* = 12, 40%), leukocytopenia (*n* = 7, 23.3%), elevated AST levels (*n* = 2, 6.7%), rash (*n* = 2, 6.7%), and proteinuria (*n* = 2, 6.7%).

**Table 3:** Summary of adverse events by severity

Adverse events	Any grade	Grade 1	Grade 2	Grade 3	Grade 4	Treatment-related grade ≥ 3
*Non-hematological toxicity*						
Increased AST level	30	23	4	3	0	2
Increased ALT level	27	23	3	1	0	1
Abnormal ECG	22	22	0	0	0	0
Vomiting	22	1	19	1	0	1
Nausea	18	15	3	0	0	0
Fatigue	18	16	2	0	0	0
Numbness	17	16	1	0	0	0
Gingivitis	15	10	5	0	0	0
Hyponatremia	15	14	0	1	0	0
Hypoproteinemia	15	10	5	0	0	0
Increased total bilirubin	13	5	5	3	0	0
Rash	11	3	6	2	0	2
Insomnia	11	5	6	0	-	0
Poor appetite	11	6	5	0	0	0
Abnormal pain	10	3	7	0	0	0
Hypopotassemia	10	8	0	2	0	0
Fever	9	2	7	0	0	0
Hoarse voice	9	9	0	0	0	0
Constipation	9	1	8	0	0	0
Epistaxis	9	9	0	0	0	0
Increases creatinine level	9	7	2	0	0	0
Hypothyroidism	8	4	4	0	0	0
Hypertension	8	0	8	0	0	0
Leg soreness	7	7	0	0	0	0
Weight loss	7	6	1	0	0	0
Headache	6	6	0	0	0	0
Hand-foot syndrome	6	4	2	0	0	0
Hyperthyroidism	5	2	3	0	0	0
Diarrhea	5	0	5	0	0	0
Cough	5	4	1	0	0	0
Tinnitus	4	4	0	0	0	0
Proteinuria	4	1	1	2	-	2
Adrenocortical insufficiency	2	1	0	1	0	1
Myocarditis	1	0	1	0	0	0
Interstitial pneumonia	1	0	0	1	0	1
Cholecystitis	1	0	1	0	0	0
Upper gastrointestinal hemorrhage	1	0	0	1	0	1
Sepsis	1	0	0	1	0	0
Gastrointestinal fistula	1	0	0	1	0	0
*Hematological toxicity*						
Thrombocytopenia	24	16	7	1	0	1
Anemic	24	16	6	2	0	1
Neutropenia	23	7	4	9	3	12
Leukocytopenia	22	3	12	7	0	7

Data are reported as no. Only AEs occurring during treatment or within 30 days of the last study medication are reported. A patient with multiple occurrences of an AE under one treatment is counted only once in the AE category for that treatment. A patient with multiple AEs is counted only once in the total row

*AEs* adverse events

### Expression of PD-L1 and whole-exome sequencing (WES)-based mutation profiling of tissues

**Fig. 3:**
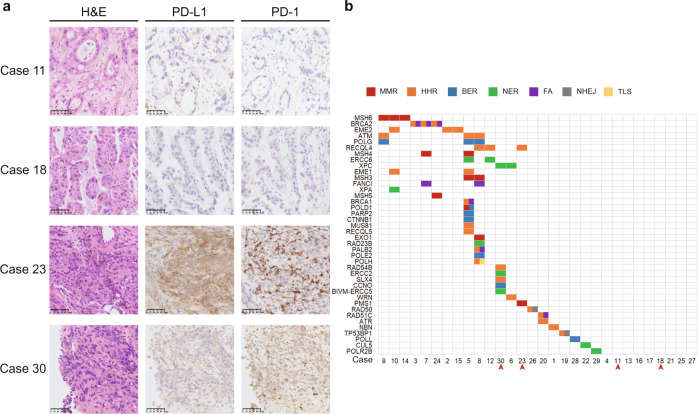
Representative case with different expression of PD-L1 protein and DDR mutation of each patient. **a** Positive PD-L1 (TAP ≥ 1%) in case 11, 23, and 30; negative PD-L1 in case 18 (TAP < 1%); Tumor area positivity (TAP) ≥ 1% was defined as positive. Proportion of tumor and/or immune cells with PD-L1 staining at any intensity. **b**. DDR-related genetic alternations and pathways identified by WES analysis in 30 patients. Red arrow refers to representative cases (case 23, 30, with multi-DDR mutation, and case 11, 18 without DDR mutation)

Immunohistochemical (IHC) examination of PD-L1 and PD-1 expression was performed. A total of 46.7% (14/30) of patients had tumors that were PD-L1 positive [tumor area positivity (TAP) ≥ 1% was defined as positive, proportion of tumor and/or immune cells with PD-L1 staining at any intensity].^8^ Representative cases with positive (cases 11, 23, and 30) and negative PD-L1 expression (case 18) are shown in Fig. 3a. After being stratified by PD-L1 expression, patients with TAP ≥ 1% tended to have a high ORR; the PD-L1-positive and -negative patient ORRs were 93% (95% CI: 66–100) and 69% (95% CI: 41–89), respectively (Supplementary Table 2).

To further investigate the molecular mechanism of action of the combined therapy in advanced ICC, WES were analyzed in 30 subjects. A total of 1564 nonsynonymous mutations were observed in 30 patients. The median tumor mutation burden (TMB) was 0.985 Muts/Mb (range, 0.08–31.82 Muts/Mb). Two patients presented with microsatellite instability high (MSI-h), and three patients had MSI-l (Table 1). *BRAF* mutations were observed in three patients, *FGFR2* fusion with *IVS15* in one subject, and an *IDH1* mutation in one subject (Table 1). Seventy percent (21/30) had DNA damage response (DDR)-related gene mutations, and 43.3% (13/30) had multi-DDR-related gene mutations (Table 1 and Fig. 3b). These mutations are involved in DDR-related pathways, including mismatch repair (MMR), nucleotide excision repair (NER), nonhomologous end joining (NHEJ), base excision repair (BER), homologous recombination repair (HRR), translesion synthesis (TLS), checkpoint factors (CPF) and Fanconi anemia (FA) (Fig. 3b). After being stratified by DDR-related gene mutations, the patients with positive DDR-related gene mutations were found to have a higher ORR than patients with negative DDR-related gene mutations [90% (95% CI: 70–99%) vs. 56% (95% CI: 21–86%), *P* = 0.049] (Supplementary Table 2). However, subgroups stratified by other baseline characteristics, including age, sex, TNM stage at enrollment, HBsAg, CA19-9 level, tumor mutation burden, and microsatellite status, presented similar ORRs (Supplementary Table 2).

## Discussion

This study was the first to demonstrate that toripalimab in combination with lenvatinib and GEMOX chemotherapy yielded a high ORR (80%) and a median OS of 22.5 months in 30 subjects with locally advanced and metastatic ICC. Grade 3 or higher AEs were observed in 17 subjects and were easily manageable.

Recently, many studies have investigated the clinical effects and safety of anti-PD-1/PD-L1 antibodies as first- and second-line therapy for BTC. A multicenter, open-label, phase 1 trial showed that nivolumab (PD-1 inhibitor) monotherapy had anti-tumor activity in Japanese patients with advanced BTC, yielding an ORR of 3.3%, a median OS of 5.2 months, and a median PFS of 1.4 months, while combination therapy of nivolumab and chemotherapy achieved more survival benefits in terms of higher ORR (33.3%), longer median OS (15.4 months), and median PFS (4.2 months).^24^ In the KEYNOTE-158 study, pembrolizumab (PD-1 inhibitor) had clinical activity in a subset of patients with unresectable or metastatic BTC, and its toxicity was manageable.^25^ Follow-up data from the KEYNOTE-028 and KEYNOTE-158 basket studies also revealed that pembrolizumab had durable clinical activity and manageable AEs in patients with locally advanced and metastatic BTC in whom standard treatment regimens had failed. The ORR in KEYNOTE-028 was 13.0%, and was 5.8% in KEYNOTE-158. The median OS in KEYNOTE-028 was 6.2 months, and the median OS in KEYNOTE-158 was 7.4 months.^26^ An open-label, single-center, three-arm, phase 2 study showed that GEMCIS chemotherapy plus durvalumab (an anti-PD-L1 antibody) with or without tremelimumab (an anti-CTLA-4 antibody) had promising antitumor activity and acceptable AEs in chemotherapy-naive patients with advanced and metastatic BTC. ORRs were 50% (15/30), 72% (34/47), and 70% (33/47) in the groups who received one cycle of GEMCIS followed by GEMCIS plus anti-PD-L1 antibody and anti-CTLA-4 antibody, GEMCIS plus anti-PD-L1 antibody, and GEMCIS plus anti-PD-L1 antibody and anti-CTLA-4 antibody, respectively.^2^ Recently, a multicenter, global, phase 3 TOPAZ-1 trial reported that GEMCIS chemotherapy plus durvalumab significantly extended median OS of 1.3 months (median OS: 12.8 vs. 11.5 months) as the first-line treatment for unresectable and metastatic BTC compared to GEMCIS chemotherapy.^8,27^ The ORR was 26.7% in the GEMCIS chemotherapy plus durvalumab group, which surpassed that in the GEMCIS chemotherapy group (18.7%).^8,27^ These data provide definite evidence for the reasonability of the combination treatment of anti-PD-1/PD-L1 antibody with chemotherapy for unresectable and metastatic BTC despite moderate OS and ORR benefits from these schemes.

BTC is, however, a heterogeneous disease; ICC, ECC, and gallbladder cancer have distinct sites of origin, molecular phenotypes, and manifestations. The data from comprehensive analysis of Advanced Biliary Tract Cancer (ABC)-01, -02, and -03 studies also showed that patients with advanced ICC or liver-only ICC had a better OS-benefit from chemotherapy compared with other BTC.^28^ Therefore, these tumors are likely to have different responses to anti-PD-1 antibody therapy. To date, only 3 studies (all case reports) have explored the efficacy of anti-PD-1 therapy on patients with pure advanced ICC.^9–11^ Combination therapy of anti-PD-1 antibody with chemotherapy or radiotherapy improved survival for all patients (*n* = 5) with advanced ICC: two achieved CR, and three achieved partial remission, with a total of decreased lesion diameters of 40.9–86.3%.^9–11^ This outcome is consistent with the current study and suggests that anti-PD-1 agents, such as toripalimab, may be useful for advanced ICC patients.

A combination therapy of lenvatinib with anti-PD-1 antibody also has utility in BTC. The LEAP-005 study recently showed that this combination yields an ORR of 10% as second-line treatment of advanced BTC.^29^ A phase 2 study with 14 advanced ICC patients showed that combining lenvatinib with an anti-PD-1 antibody (pembrolizumab or nivolumab) yielded an ORR of 21.4% and a DCR of 92.9%. The median PFS was 5.9 months.^30^ Our other two-cohort, single-center, phase 2 study investigated the efficacy and safety of lenvatinib in combination with toripalimab or GEMOX chemotherapy for systemic treatment-naive patients with advanced or unresectable ICC (NCT04361331). Our results showed that toripalimab combined with lenvatinib yielded an ORR of 32.3% (10/31, 95% CI: 16.6–51.4%) and DCR of 74.2% (23/31, 95% CI: 55.4–88.1%). Combination therapy of GEMOX with lenvatinib for systemic treatment-naive patients with advanced ICC yielded an ORR of 30% (10/30) and DCR of 86.7% (26/30).^31,32^ Our current study focused on the synergistic efficacy of combining all three treatments (immune checkpoint inhibitor anti-PD-1 antibody, tyrosine kinase inhibitor lenvatinib, and GEMOX chemotherapy) for pure advanced ICC. We found an ORR of 80%, which is much higher than those of the pairwise combinations for ICC^31,32^ and the combination treatment of GEMOX with anti-PD-1 antibody for advanced BTC.^33^ This suggests that toripalimab, lenvatinib, and GEMOX may have additive and/or synergistic inhibitory effects on cholangiocarcinoma cells.

In addition to being beneficial, our triple treatments resulted in AEs that were tolerable and manageable. The common AEs observed in this study were increased AST or ALT levels, thrombocytopenia, anemia, neutropenia, leukocytopenia, abnormal ECG, vomiting, nausea, and fatigue, which were similar to those reported in a previous study of chemotherapy and an anti-PD-1 antibody.^8^ Hematologic toxicity was the main grade 3 or higher AE in this study as well as the most common TEAE for toripalimab in a phase 3 study on advanced nasopharyngeal carcinoma.^34^ In the present study, dose modification was observed in six subjects and medication delay in ten subjects due to AEs. However, there was no discontinuation or death due to treatment-related AEs. Based on the above data of controllable AEs, this combination therapy is expected to be safe in ICC patients with ECOG 0/1 status, but this needs to be further validated clinical trials in future.

All patients with advanced ICC included in this study had stage IIIA disease or higher at baseline: 16.7% (5/30) had stage IIIA disease, 43.3% (13/30) had stage IIIB disease, and 40% (12/30) had stage IV disease. This means that the severity of the disease in the present study was similar to that in the ABC-02 trial (104/410 patients had locally advanced disease and 306/410 had metastases), which tested chemotherapy alone. Our combined therapy resulted in a much higher ORR and better mOS for advanced ICC than standard chemotherapy or any pairwise combination of PD-1 inhibitor with lenvatinib or chemotherapy. Moreover, this study showed that patients with positive staining for PD-L1in tumor cells tended to have a good response to this triple combination therapy. More importantly, our results also showed that patients presented with DDR-related gene mutations had a better response to this regimen than those without mutations. DDR is an important mechanism that enables cell survival in the face of genomic instability, replicative stress, and irreparable damage.^35^ Recently, the vulnerabilities of cancer cells owing to defects in DDR pathways have been exploited in anticancer therapy with DNA- damaging radiation and chemotherapies and with DDR inhibitors.^35,36^ In the present study, this triple-combination therapy was more effective in patients presented with DDR-related gene mutations. DDR-related gene mutation may become a good predictor of the tumor response to this regime of advanced ICC.

This study has a few limitations. First, all patients were from a single center, potentially introducing an unknown selection bias and reducing the generalizability of the study. In particular, only relatively young patients (median 56.5 y, range 25–73 y) and those with good performance status were enrolled in this study, which may result in selection bias. Second, this was a single-arm, single-center and phase 2 trial and was designed without a control, which might weaken the reliability of evidence and increase the comparison error. Finally, the sample size in present trial was relatively small, which may result in selection bias and influence the estimation of ORR, PFS, and OS.

Despite these limitations, this phase 2 study is, to the best of our knowledge, the first prospective trial of a PD1 inhibitor combined with GEMOX chemotherapy and lenvatinib for pure and advanced ICC patients. This study provides believable data demonstrating high efficacy, controllable AEs, and feasibility of this triple-combination therapy for ICC, which should be further explored in future well-designed prospective trials. To that end, we recently have developed a multicenter, double-blinded, randomized, phase 3 study to confirm the high efficacy of this combination therapy in patients with advanced ICC, and obtained NMPA approval (No 2021LP01825). That trial is also registered at Clinicaltrials.gov (number NCT05342194). It is expected that this new study will start to enroll patients in a few months.

## Materials and methods

### Study design and participants

A single-center, single-arm, open-label, phase 2 study was designed to evaluate the efficacy and safety of toripalimab in combination with lenvatinib and GEMOX chemotherapy as first-line therapy for unresectable and metastatic ICC in Zhongshan Hospital. Forty-two candidates were screened, and 30 eligible subjects were enrolled in this trial from May 15, 2019, to October 24, 2019 (Fig. 1). The main inclusion criteria were as follows: 18–75 years old, histopathologically confirmed advanced and systemic treatment-naive ICC with measurable lesion per RECIST 1.1, Eastern Cooperative Oncology Group (ECOG) performance status score 0, and Child–Pugh classification A. Functional indicator requirements were as follows: (1) absolute neutrophil count (ANC) ≥ 1.5 × 10^9^/L, platelets ≥ 100 × 10^9^/L, hemoglobin ≥ 9.0 g/dL, and serum albumin ≥ 3.0 g/dL; (2) thyroid-stimulating hormone (TSH) ≤ 1.0 time the upper limit of the normal range and T3 and T4 within the normal ranges; (3) total bilirubin ≤1.5 times upper limit of normal (ULN) and serum alanine aminotransferase (ALT) and aspartate aminotransferase (AST) ≤ 1.5 times ULN; and (4) serum creatinine ≤ 1.5 times ULN and creatinine clearance ≥60 mL/min. The main exclusion criteria were as follows: hepatocellular carcinoma or combined hepatocellular-cholangiocarcinoma; active infection, infectious diseases, autoimmune diseases, or other diseases requiring long-term hormones or immunosuppressive therapy; severe cardiopulmonary and renal dysfunction or uncontrolled hypertension; HBV-DNA > 2000 copies/mL; prothrombin time (PT) > 14 s, receiving thrombolysis or anticoagulation therapy or having a bleeding tendency within 3 months before enrollment; history of platinum allergy or previous treatment with anti-PD-1/PD-L1 antibody, or anti-CTLA-4 antibody; psychotropic drug use, alcohol use, or drug abuse; or serious mental illness or laboratory abnormalities.

GEMOX chemotherapy is a common therapeutic schedule for advanced BTC, including ICC. The safety of combination treatment of lenvatinib with anti-PD1 antibody for patients with advanced ICC was acceptable.^30^ Thus, the GEMOX regimen in combination with 8 mg lenvatinib and toripalimab was theoretically safe for patients with ICC and adequately functioning major organs, especially the liver. Advanced ICC is a highly lethal disease with very short expected OS and needs to be effectively intervened against as early as possible.

The research protocol was approved by the Zhongshan Hospital review board and independent ethics committees (B2019-078R) and was conducted following the Declaration of Helsinki and Good Clinical Practice Guidelines. Each subject was required to sign the informed consent form before enrollment. WES of DNA from fresh biopsy tumor tissues was performed as reported,^37^ and IHC was used to detect PD-L1 and PD-1 protein expression in paraffin-embedded tumor tissues.^14^ Common mutations, e.g., BRAF and IDH1 mutations, DDR-related gene mutations, microsatellite instabilities, and FGFR fusions and rearrangements, were explored.

### Procedures

Eligible subjects received intravenous GEMOX chemotherapy (Q3W) including oxaliplatin (85 mg/m^2^) on day 1, and gemcitabine (1 g/m^2^) on days 1 and 8 for 6 cycles. They also received intravenous toripalimab (240 mg, Q3W) on day 1 and oral lenvatinib (8 mg, once daily) for one year. After one year, a continuing treatment plan was determined via consultation between the researcher and the patient. Patients with CR, PR, or SD continued to receive toripalimab and lenvatinib, and patients with PD received best supportive care or local traditional treatment, including FOLFIRI (5-fluorouracil, folinic acid and irinotecan), reuse of GEMOX, and regorafenib. Surgery was permitted for patients who achieved a PR suitable for R0 resection. Treatment was discontinued at the time of PD, intolerable toxicity, investigator judgment, or withdrawal of the informed consent of the subject. Tumor responses were evaluated using enhanced computed tomography or magnetic resonance imaging every 9 weeks (range 8–10 weeks) by the investigator. The independent post hoc analysis followed RECIST 1.1. Physical examinations and laboratory evaluations were conducted to assess the safety before each cycle of GEMOX infusion and/or every dose of toripalimab. Dose modifications of GEMOX and lenvatinib were allowed to manage TEAEs. AEs were coded following the Common Terminology Criteria for AEs, version 5.0 (CTCAE V5.0).

### Outcomes and assessments

The primary endpoint of this trial was ORR, including complete and partial responses as determined by the investigator and independent post hoc analysis. Secondary endpoints included OS (dated from the first treatment with study medication to the date of death owing to any cause or censored on the date of last follow-up), PFS (dated from the first treatment with study medication to the first documented PD or death owing to any cause, whichever occurred first; defined as tumor recurrence or death owing to any cause in the patients who received resection), 1-year overall survival rate, DoR, and DCR (including CR, PR, and SD). These endpoints were inputs into the efficacy and safety profiles assayed by the investigators or by the two independent radiologists in the post hoc analysis.

### IHC analysis and WES analysis

Tumor tissues of each patient were obtained by biopsy and embedded by paraffin. The presence of PD-L1 and PD-1 was investigated by IHC staining with corresponding primary antibody as previously.^14^ Tumor area positivity (TAP) was defined as the percentage of PD-L1-positive cells (including PD-L1-positive tumor and/or immune cells) in total cells. TAP ≥ 1% was defined as positive.^8^

DNA in paraffin-embedded biopsy tissues from each subject was extracted. Sequencing libraries were prepared and whole-exome-sequenced according to the instructions of manufacturer. The raw data of WES have been uploaded in the OMIX, China National Center for Bioinformation/Beijing Institute of Genomics, Chinese Academy of Sciences (https://ngdc.cncb.ac.cn/omix: accession no.OMIX002452).

Mutation calling by Trimmomatic was used as quality control of FASTQ. Paired-end reads were then aligned to the human reference genome (hg19) using the Burrows–Wheeler aligner (BWA). Somatic mutations were first acquired for each sample. All single nucleotide variants (SNVs)/indels were annotated by ANNOVAR, and each SNV/indel was manually examined in the Integrative Genomics Viewer (IGV). Copy number variations (CNVs) were analyzed using in-house software. Mutations in 15 genes related to microsatellites (MS) were analyzed, and ≥30% of mutations were defined as MSI-h. Mutations in DDR-related genes were analyzed.

### Statistical analysis

The sample size of this trial was calculated according to an exact single-stage design. A total ORR ≤ 14% (the ORR was 13.5% in our previous observational study on GEMOX for advanced ICC, unpublished data) was considered not clinically meaningful; an overall response ≥40% was considered promising. Thirty patients were needed to achieve a power of 90% with a significant level of 5%. All efficacy and safety endpoints were analyzed in all subjects who obtained at least one dose of toripalimab.

The ORRs and 95% confidence intervals (CIs) were determined by the Clopper-Pearson method. We calculated the number of subjects with a best tumor response and the DCR. We calculated the maximum percentage change in the sum of the diameters of the target lesions for each subject (from baseline) and pictured these parameters as a waterfall plot. We used the Kaplan‒Meier method to summarize OS, PFS, and DoR. Medians and 95% CIs (calculated with the Brookmeyer-Crowley method) are presented. Further, 95% CIs for OS and PFS rates were determined based on the Greenwood formula. Post hoc ORR subgroup analyses stratified by selected demographic and disease characteristics were conducted accordingly. Exploratory subgroup analysis stratified by intensity of tumor PD-L1 and DDR-related gene mutation was performed.

All data were analyzed by the aid of SPSS 19.0 software (IBM, Armonk, NY, USA) and R software version 4.1.0 (The R Foundation, Vienna, Austria). This trial is registered with Clinicaltrials.gov, number NCT03951597.

## Supplementary information

supplemental table

## Data Availability

The dataset generated during the present study can be obtained from the corresponding author with a reasonable request.
