# Supplementary material for: Toripalimab combined with lenvatinib and GEMOX is a promising regimen as first-line treatment for advanced intrahepatic cholangiocarcinoma: a single-center, single-arm, phase 2 study
Source: Signal Transduct Target Ther. 2023 Mar 17;8:106. doi: 10.1038/s41392-023-01317-7 (PMC10020443; doi:10.1038/s41392-023-01317-7)
Supplement: Supplementary file 1 — supplemental table [file 41392_2023_1317_MOESM1_ESM.docx]

Supplementary Materials for

**Toripalimab combined with lenvatinib and GEMOX is a promising regimen as first-line treatment for advanced intrahepatic cholangiocarcinoma: A single-center, single-arm, phase 2 study**

Guo-Ming Shi^1, ¶^, Xiao-Yong Huang^1, ¶^, Dong Wu ^2, ¶^, Hui-Chuan Sun^1, ¶^., Fei Liang^3^, Yuan Ji^4^, Yi Chen^5^, Guo-Huan Yang^1^, Jia-Cheng Lu^1^, Xian-Long Meng^1^, Xin-Ying Wang^6^, Lei Sun^6^, Ning-Ling Ge^5^, Xiao-Wu Huang^1^, Shuang-Jian Qiu^1^, Xin-Rong Yang ^1^, Qiang Gao^1^, Yi-Feng He^1^, Yang Xu^1^, Jian Sun^1^, Zheng-Gang Ren^5^, Jia Fan^1, *^, Jian Zhou^1, *^

Correspondence to: [zhou.jian@zs-hospital.sh.cn](mailto:zhou.jian@zs-hospital.sh.cn)

or [fan.jia@zs-hospital.sh.cn](mailto:fan.jia@zs-hospital.sh.cn)

**This PDF file includes:**

Tables S1 to S2

Table S1.

**Best Response of Each Patient to Treatment**

| **Subject no.** | **Tumor response** | **DoR (months)** | **TTR (months)** | **TNM stage** |
| --- | --- | --- | --- | --- |
| 5 | CR | 30.5 | 2.1 | IV |
| 2 | PR | 12.5 | 2 | IIIA |
| 12 | PR | 6.2 | 2.1 | IIIA |
| 19 | PR | 13.8 | 2.1 | IIIA |
| 22 | PR | 23.7 | 6.4 | IIIA |
| 1 | PR | 7 | 2.3 | IIIB |
| 3 | PR | 18.1 | 2.1 | IIIB |
| 6 | PR | 13.6 | 4.3 | IIIB |
| 10 | PR | 8.4 | 3.3 | IIIB |
| 13 | PR | 3.4 | 4.2 | IIIB |
| 17 | PR | 9.8 | 2.1 | IIIB |
| 20 | PR | 4.3 | 2.3 | IIIB |
| 27 | PR | 2.5 | 2.0 | IIIB |
| 28 | PR | 15.0 | 2.1 | IIIB |
| 29 | PR | 12.2 | 2.1 | IIIB |
| 30 | PR | 30.6 | 2.1 | IIIB |
| 4 | PR | 7.8 | 2.1 | IV |
| 7 | PR | 6.9 | 2.0 | IV |
| 9 | PR | 5.1 | 2.1 | IV |
| 14 | PR | 15.2 | 2.5 | IV |
| 15 | PR | 21.9 | 2.0 | IV |
| 16 | PR | 14.7 | 2.1 | IV |
| 23 | PR | 7.5 | 2.0 | IV |
| 24 | PR | 8.1 | 2.1 | IV |
| 18 | SD |  |  | IIIA |
| 21 | SD |  |  | IIIB |
| 25 | SD |  |  | IV |
| 26 | SD |  |  | IV |
| 11 | PD |  |  | IIIB |
| 8 | NE |  |  | IV |

**Appreciation:** NE, not evaluated; CR, complete response; PR, partial response; SD, stable disease; PD, progressive disease; TTR, Time to response; DoR, Duration of response; TNM, tumor nodule metastasis.

Table S2.

**ORR of Patients in the Subgroup According to Baseline Characteristics**

| **Subgroup** | **No. of PR or CR/total no. of patients** | **ORR, %**  **(95% CI)** |
| --- | --- | --- |
| ALL | 24/30 | 80 (61–92) |
| Age |  |  |
| <65 years | 14/19 | 74 (49–91) |
| ≥65 years | 10/11 | 91 (59–100) |
| Sex |  |  |
| Male | 15/19 | 79 (54–94) |
| Female | 9/11 | 82 (48–98) |
| TNM stage at baseline |  |  |
| IIIA | 4/5 | 80 (28–99) |
| IIIB | 11/13 | 85 (55–98) |
| IV | 9/12 | 75 (43–95) |
| History of hepatolithiasis |  |  |
| Yes | 1/1 | 100 (2.5–100) |
| No | 23/29 | 79 (60–92) |
| HBsAg |  |  |
| Positive | 7/8 | 87.5(47–100) |
| Negative | 17/22 | 77.3 (55–92) |
| HBcAb |  |  |
| Positive | 19/24 | 79 (58–93) |
| Negative | 5/6 | 83 (36–100) |
| PD-L1 expression (IHC ≥1%*) |  |  |
| Positive | 13/14 | 93 (66–100) |
| Negative | 11/16 | 69 (41–89) |
| CA19-9 (U/mL) |  |  |
| ≥37 | 16/21 | 76 (53–92) |
| <37 | 8/9 | 89 (52–100) |
| Tumor mutation burden (Muts/Mb) |  |  |
| High (≥0.985) | 11/15 | 73 (45–92) |
| Low (<0.985) | 13/15 | 87 (60–98) |
| DDR-related gene mutation |  |  |
| Present | 19/21 | 90 (70–99) |
| Absent | 5/9 | 56 (21–86) |
| MSI status |  |  |
| High | 1/2 | 50(1-99) |
| Low | 3/3 | 100(29-100) |
| Stable | 20/25 | 80(59-93) |

**Appreciation**: HBsAg, hepatitis B surface antigen; HBcAb, hepatitis B core antibody; PD-L1, programmed cell death-ligand 1; CA19-9, carbohydrate antigen 19-9; DDR, DNA damage response; MSI, microsatellite instability.

* Tumor area positivity (TAP) ≥ 1% was defined as positive. Proportion of tumor and/or immune cells with PD-L1 staining at any intensity.
